# Pivotal role of intestinal cholesterol and nuclear receptor LXR in metabolic liver steatohepatitis and hepatocarcinoma

**DOI:** 10.1186/s13578-024-01248-y

**Published:** 2024-06-01

**Authors:** Elena Piccinin, Maria Arconzo, Emanuela Pasculli, Angela Fulvia Tricase, Silvia Cultrera, Justine Bertrand-Michel, Nicolas Loiseau, Gaetano Villani, Hervé Guillou, Antonio Moschetta

**Affiliations:** 1 Department of Interdisciplinary Medicine, University of Bari “Aldo Moro”, Piazza Giulio Cesare 11, Bari, 70124 Italy; 2 Department of Translational Biomedicine and Neuroscience (DiBraiN), University of Bari “Aldo Moro”, Bari, Italy; 3 Toxalim (Research Center in Food Toxicology), INRAE, ENVT, INP-PURPAN, UMR 1331, UPS, Université de Toulouse, Toulouse, France; 4 MetaboHUB-MetaToul, National Infrastructure of Metabolomics and Fluxomics, Toulouse, France; 5 INBB, National Institute for Biostructures and Biosystems, Rome, Italy

**Keywords:** Non-alcoholic steatohepatitis, Hepatocellular carcinoma, Gut-liver axis, Lipid metabolism, Liver X receptor

## Abstract

Hepatocellular carcinoma (HCC) incidence is continuously increasing worldwide, due to the rise of metabolic dysfunction-associated steatohepatitis (MASH) cases. Cholesterol is an essential driver of the metabolic dysregulations that promote HCC progression. Liver X Receptor (LXR) is a nuclear receptor best known for the regulation of lipid and cholesterol homeostasis, with a prominent function in the liver and in the intestine. Here, we aimed to explore whether modifications in intestinal lipid metabolism may contribute to the onset of HCC, particularly taking into account cholesterol metabolism and LXRs. To study the progression of MASH to HCC, we induced metabolic HCC in wild-type male mice and mice carrying an intestinal chronic activation of LXRα. Also, we analysed human hepatic transcriptome datasets. The increased consumption of fat and carbohydrates drives the intestinal activation of LXRα and accelerates the onset of the hepatic tumours. Chronic intestinal-specific activation of LXRα enhances HCC progression only in the presence of a high cholesterol intake. In HCC, despite the increased hepatic cholesterol content, LXR is not active, thus driving liver cancer development. Intriguingly, in line with these results in the mouse model, LXR transcriptome is also downregulated in human hepatocarcinoma and its expression level in liver tumours directly correlates with a decreased survival rate in patients. Overall, our findings establish the relevance of the intestine in influencing the susceptibility to MASH-HCC and point to intestinal LXRα activation as a driver of metabolic liver cancer in the presence of dietary cholesterol.

**Supplementary Information:**

The online version contains supplementary material available at 10.1186/s13578-024-01248-y.

## Introduction

Hepatocellular carcinoma (HCC) is the fifth most common malignancy worldwide, with a rising incidence, a high mortality rate and a poor prognosis due to limited therapeutic options. The pathophysiology of HCC is a complex multistep process, involving chronic hepatitis and fibrosis, finally leading to overt liver cancer [1]. In Western Countries, metabolic dysfunction-associated steatohepatitis (MASH), together with metabolic syndrome and related comorbidities, is now considered the fastest-growing aetiology of the burden of HCC [2].

Metabolic reprogramming represents one of the critical hallmarks of cancer pathogenesis, characterised by alterations of different molecular mechanisms to fuel cell growth and division, and changes in lipid metabolism are widely involved in hepatocarcinogenesis and tumour adaptation to various conditions [3].

Cholesterol plays a pivotal role in cell proliferation in physiological and tumoral states. Since both cholesterol accumulation and depletion can be deleterious, different mechanisms to control cholesterol homeostasis within the cell have been developed. When the cholesterol levels are low, the hepatocyte copes by increasing the uptake of cholesterol via the Low-Density Lipoprotein Receptor (*LDLR*) and the Niemann-Pick C1-Like 1 (*NPC1L1*), or by increasing the synthesis of new cholesterol coordinated by the Sterol regulatory element-binding protein 2 *(SREBP2)* [4, 5]. By contrast, the cells promptly convert high cholesterol into cholesterol derivatives, such as cholesteryl esters and oxysterols. The latter are the ligands of the Liver X Receptors (LXRs), which act as master regulators of cholesterol homeostasis by upregulating the expression of genes involved in cholesterol excretion, including the ATP Binding Cassette 5 and 8 (*ABCG5/8*) [6, 7]. Of note, in the liver, the activation of LXRs also boosts the synthesis of new fatty acids, acting directly on the Sterol regulatory element-binding protein 1c *(SREBP1c)* and major de novo lipogenesis genes, Fatty Acids Synthase (*FASN*) and Stearoyl-CoA desaturase-1 (*SCD1*) [8].

LXRs family comprised two different isoforms, LXRα (*NR1H3*) and LXRβ (*NR1H2*), both ubiquitously expressed, with a peak of expression of LXRα in the liver, intestine, adipose tissue and macrophages [9]. The literature has widely investigated the antitumoral effect of LXRs’ activation on breast [10], colon [11] and prostate cancer [12] as well as melanoma [13]. More specifically, LXRs’ activation causes a shift from anabolic to catabolic pathways, thus limiting the intracellular cholesterol content accumulation [14]. During the rapid growth of cancer cells, there is a need for intracellular cholesterol disposal to sustain the high growth rate. This process is accompanied by a decrease in oxysterol content, which results in the downregulation of the LXRs transcriptome. Specifically, this net uncoupling between oxysterols and cholesterol levels is characterized by the downregulation of oxysterol synthesis and the simultaneous induction of catabolic and secretory pathways [14].

In HCC, the role of LXRs is still controversial: while the usage of LXR agonists has been correlated with increased lipotoxicity and limited tumour growth [15–17], the chronic activation of LXRs promotes HCC in mice [18].

Our group has previously generated the iVP16LXRα mouse models, in which the expression of the human isoform of LXRα is selectively and constitutively expressed in the intestine. Taking advantage of this model, we demonstrated that the chronic activation of intestinal LXRs limits cholesterol absorption, concomitantly increasing reverse cholesterol transport with protection against atherosclerosis [19]. Whether the intestinal activation of LXRs may play a role in HCC development has not been investigated so far. Since a high-fat diet per se is not sufficient to drive inflammation and fibrosis, and cholesterol has to be present to generate a MASH-driving HCC condition [20], we wonder if a Western diet (WD) enriched in cholesterol exerts any effect on the hepatic LXRs in HCC and if the intestine plays a role in this scenario.

In this study, to resemble the human state, we combined the hepatic carcinogen to a diet that drives metabolic conditions and we uncovered an unexpected role of intestinal LXRs in HCC onset in association with dysmetabolic settings. Altogether, our results point to lipids as the crucial drivers of MASH and liver cancer.

## Materials and methods

### Animals

Mice were kept in a pathogen-free facility, at 21 ± 2 °C with a 12 h light/dark cycle and had free access to food and water. All the murine strains we used were in C57BL6/J background. Previously described iVP16LXRα mice and iVP16 controls [19] were backcrossed with wildtype C57BL6/J mice for more than 10 generations to obtain a C57BL6/J pure background. Regardless of genotype, fifteen-day-old male mice were intraperitoneally injected with a single dose of diethyl-nitrosamine (DEN) and then randomly assigned either to a chow diet Chow diet (Global diet 2018, Teklad) or a western diet (D12079B, Research Diet) feeding for 8–12 months. About 7–12 animals per group were used. Food intake and body weight were monitored weekly. All mice were sacrificed randomly after 4 h of fasting at ZT6. Mice experiments were performed according to the ethical protocol authorized by the Italian Ministry of Health (n.1049/2020-PR).

### Organs and blood sampling

At the time of sacrifice, the liver was removed, and weight and tumours were counted and measured. All the tissues were removed, dissected, snap-frozen in liquid nitrogen and stored at -80 °C until use, or immersed in formalin for IHC analyses. For the ileum section, intestinal cells were collected by scraping, snap-frozen in liquid nitrogen, and stored at -80 °C until use. Blood was collected by cardiac puncture using heparin-coated syringes. Plasma was prepared by centrifugation (1000×g, 10 min at 4 °C) and kept at -80 °C until use.

### Histology and immunohistochemistry

Tissue specimens harvested from mice were fixed in 10% formalin for 12–24 h, dehydrated, and paraffin-embedded. Ileum and liver Sect. (2 μm) were stained with haematoxylin-eosin staining (HE), according to the standard procedures. Sirius Red staining using Direct Red 80 and Fast Green FCF (Sigma-Aldrich, USA) was performed on liver sections to assess fibrosis. Immunohistochemistry analysis was performed in liver specimens (4 μm). Briefly, sections were subjected to antigen retrieval by boiling the slides in sodium citrate pH 6 for 15 min, permeabilized in phosphate-buffered saline with 0.25% Triton X-100 for 5 min, and then incubated for 10 min at room temperature in protein blocking solution (Dako, Denmark). Subsequently, sections were incubated with primary antibodies (Table 1). Sections were washed in PBS for 15 min and incubated at room temperature with DAKO real EnVision detection system (Dako, Denmark), according to the manufacturer’s instruction. For negative controls, 1% nonimmune serum in PBS substituted the primary antibodies. Images were acquired and analysed with Aperio Image Scope (Leica Biosystems, Germany). The percentage of stained area/total area was evaluated in 10 consecutive acquired images. Values from all consecutive images for each sample were averaged and displayed as mean ± SEM.

**Table 1:** List of antibodies used in the study

Antibody	Company	Catalogue	Host	Dilution
Ki67	Abcam	ab15580	Rabbit	1:200
F4/80	Cell Signaling	#70076	Rabbit	1:200

### Gene expression

For the liver, the intestinal tracts, the gastrocnemius, and the ileum scraping harvested from mice total RNA was extracted with Qiazol reagent (Qiagen, Germany). 1–2 µg of total RNA was treated with DNase (Thermo Fisher Scientific, USA) and retrotranscribed to cDNA using the High Capacity cDNA Reverse Transcription Kit (Thermo Fisher Scientific, Massachusetts, USA) following the manufacturer’s instructions. qPCR assays were performed in 96-well plates using the Master Mix Power SYBR Green (Thermo Fisher Scientific, USA) via the QuantStudio5 machine (Thermo Fisher Scientific, USA), and the first analysis was performed using the QuantStudio Design & Analysis. Relative quantification was calculated via the ΔΔCT method, using Tbp (TATA-binding protein) as a reference gene. All the primers used in this study are listed in Table 2.

**Table 2:** List of primers used in the study

Gene	Sequence Primer Forward	Sequence Primer Reverse
Abcc1	CAGTGGTTCAGGGAAGGAGTCA	CACTGTGGGAAGACGAGTTGCT
Abcg5	TCAATGAGTTTTACGGCCTGAA	GCACATCGGGTGATTTAGCA
Abcg8	AATGTCATCCTGGATGTCGTCTC	CCAGCTCATAGTACAGCATTGACC
Acta2	GTTCAGTGGTGCCTCTGTCA	ACTGGGACGACATGGAAAAG
Ccnd1	CATCCATGCGGAAAATCGT	TCTACGCACTTCTGCTCCTCA
Ccne1	GCTTCTGCTTTGTATCATTTCTCCTC	GGAACCATCCATTTGACACACTT
Col1a1	TAGGCCATTGTGTATGCAGC	ACATGTTCAGCTTTGTGGACC
Cyp27a1	GCCTCACCTATGGGATCTTCA	TCAAAGCCTGACGCAGAGT
Cyp46a1	GCGCGCTTCAGACTGTGTT	GCGCCCATAGTCACATTCAG
Fasn	AGTCAGCTATGAAGCAATTGTGGA	CACCCAGACGCCAGTGTTC
Hmgcr	CTTGTGGAATGCCTTGTGATTG	AGCCGAAGCAGCACATGAT
Hmgcs	TGCAGGAAACTTCGCTCACA	TGCAGGAAACTTCGCTCACA
Il6	GTATGAACAACGATGATGCACTTG	ATGGTACTCCAGAAGACCAGAGGA
Ldlr	AGGCTGTGGGCTCCATAGG	TGCGGTCCAGGGTCATCT
Lxrβ	AAGCAGGTGCCAGGGTTCT	TGCATTCTGTCTCGTGGTTGT
Mmp9	CTCGAGGGCTTCCCTCTGA	GGCTGGAGGCCTTGGGT
Mvk	GCTTCAGCGACTGGACACG	ACAGGTAGAGAAAGGCAAGCAGA
Npc1l1	AGATCCCAACTTTGAGGTCTTCC	ACCGTCAGGTATTGCTGGTAGAAC
Nr1h3	AGGAGTGTCGACTTCGCAAA	CTCTTCTTGCCGCTTCAGTTT
Scarb1	TCCCCATGAACTGTTCTGTGAA	TGCCCGATGCCCTTGA
Scd1	CAGTGCCGCGCATCTCTAT	CAGCGGTACTCACTGGCAGA
Sqle	GGAGGCTACCGTGTTCTCCA	CTGCACTTGGTTGGTTTCTGAC
Srebp1c	GGAGCCATGGATTGCACATT	GGCCCGGGAAGTCACTGT
Srebp2	GCGTTCTGGAGACCATGGA	ACAAAGTTGCTCTGAAAACAAATCA
Sult2b1	CGCCCTGTGGAGCTCGT	TGAGGCTCTCCGGTGAGTACAT
Tgfb	GCAGTGGCTGAACCAAGGA	AGAGCAGTGAGCGCTGAATC
Tnfa	CTGAGGTCAATCTGCCCAAGTAC	CTTCACACAGCAATGACTCCAAAG

### Lipidomic Assay

Neutral lipids were extracted from plasma, liver or intestine frozen tissues harvested from mice using a Bligh and Dyer extraction method: samples were homogenized in methanol/5 mM EGTA (2:1, v/v), and lipids (corresponding to an equivalent of 2 mg tissue) extracted according to the Bligh–Dyer method, with chloroform/methanol/water (2.5:2.5:2 v/v/v), in the presence of the following internal standards: glyceryl trinonadecanoate, stigmasterol, and cholesteryl heptadecanoate (Sigma-Aldrich). Triglycerides, free cholesterol, and cholesterol esters were analysed by gas-liquid chromatography on a Focus Termo Electron system equipped with a Zebron- 1 Phenomenex fused-silica capillary column (5 m, 0.25 mm i.d., 0.25 mm film thickness). The oven temperature was programmed to increase from 200 to 350 °C at 5 °C/min, and the carrier gas was hydrogen (0.5 bar). The injector and detector temperatures were 315 °C and 345 °C, respectively. Fatty acids were extracted from frozen tissues or plasma using a modified Bligh and Dyer extraction method. Samples were lysed in a water EDTA (5 Mm)/methanol mix (1:2 vol/vol). Methanol and dichloromethane were added to reach the following ratios of MeOH/water/CH2Cl2: 2.5/2.0/2.5. Glyceryltrinonadecanoate was added as an internal standard. The dried lipid extract was transmethylated with 1 ml of BF3 in methanol (1:20, vol/vol) for 60 min at 100 °C, evaporated to dryness, and the fatty acid methyl esters (FAMEs) were extracted with hexane/water (3:1). The organic phase was evaporated to dryness and dissolved in 50 µl ethyl acetate. FAMEs were analyzed by gas-liquid chromatography on a 5890 Hewlett-Packard system using a Famewax fused-silica capillary column (30 m, 0.32 mm i.d., 0.25-mm film thickness; Restek). The oven temperature was programmed from 110ºC to 220 °C at a rate of 2 °C/min, and the carrier gas was hydrogen (0.5 bar). The injector and the detector were at 225ºC and 245 °C, respectively.

### Human dataset analysis

Expression of LXR, ABCG5 and ABCG8 in human HCC samples was obtained and explored in-silico from TCGA TARGET GTEx cohort using UCSC Xena browser (http://xena.ucsc.edu), and replotted using GraphPad Software. Genes-level quantification data is reported in log2 transformed normalized expression counts. All clinical metadata was obtained from the TCGA TARGET GTEx file on the Xena browser’s Phenotypes page for the dataset TCGA. High, middle and low expression were obtained by dividing samples into the upper third, middle third, and lower third. GEO datasets were selected according to the following inclusion parameters: (1) histological diagnosis; (2) hepatocellular carcinoma or liver tumour for the experimental group; (3) normal or adjacent non-tumoral tissue used as controls; (4) RNA profiling by array and raw data required the CEL or TXT format; (5) Homo sapiens specimens. We used the search terms hepatocellular carcinoma OR hcc OR liver tumour AND Homo Sapiens AND Expression profile by the array in the GEO DataSets to identify potential datasets. Then, we further screened these datasets according to the following exclusion criteria: datasets with specimens from other organisms or cell lines, expression profiling by RT-PCR or RNA sequencing, sample size < 125, not including the gene of interest (NR1H3, ABCG5, ABCG8), not including controls or controls coming from intestinal cancer patients, specimens from HCV/HBV patients or patients under treatment/alcohol, specimens from FFPE. Finally, 4 GEO datasets, GSE54236, GSE57957, GSE64041 and GSE112790, were included in our study.

### Statistics

All the results are expressed as mean ± SEM. Statistical analyses were performed with GraphPad Prism software analysis (v9.0, GraphPad Software, USA). Outliers were calculated with the ROUT test. To compare two groups the Whitney U test was used. A p-value < 0.05 was considered significant. Significant effect was indicated by * (*p < 0.05, **p < 0.01, ***p < 0.001).

### Access to Data

All authors had access to the all data and have reviewed and approved the final manuscript.

## Results

*Western diet feeding promotes liver tumours in mice.*

**Fig. 1:**
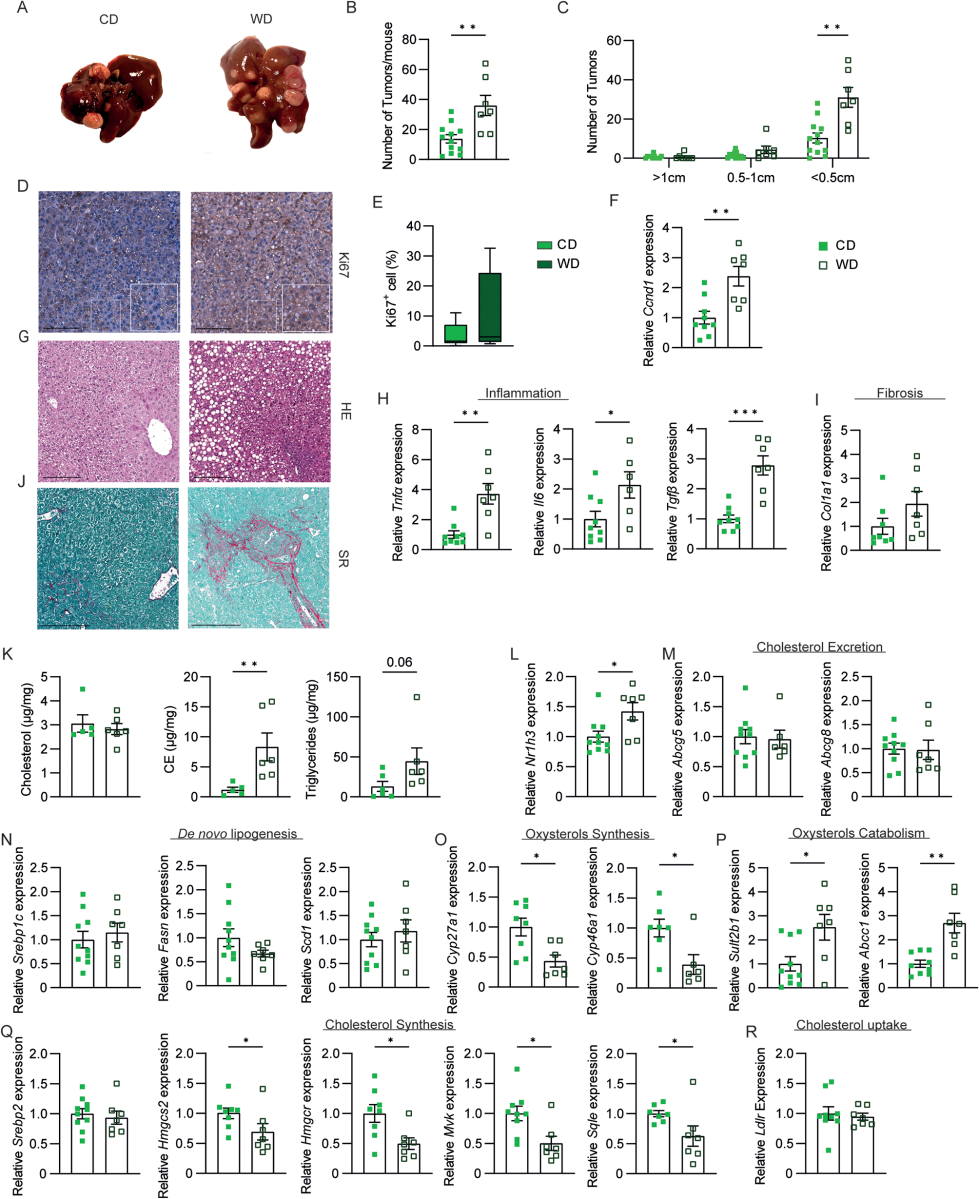
**Western diet feeding promotes liver tumours in mice.** Fifteen-day-old Wild Type C57BL6/J mice were injected with diethyl-nitrosamine (DEN) and then fed either a chow diet (CD) or a Western diet (WD) starting from one month of age. Mice were sacrificed after 12 months (*n* = 7–12 animals/group). (A) Gross morphology of the liver at the moment of sacrifice. (B) The average number of tumours per mouse. (C) The average number of tumours per mouse based on tumour diameter cut-off. (D) Ki67 staining and (E) quantification (scale bar 200 μm) of tumoral liver section. (F) Hepatic relative mRNA expression of Cyclin D1 (*Ccnd1*) in liver tumour specimens. (G) Haematoxylin-Eosin (HE) of tumoral liver section (scale bar 200 μm). Hepatic relative mRNA expression of genes involved in (H) inflammation and (I) fibrosis. (J) Sirius Red staining of tumoral liver section (scale bar 200 μm). (K) Hepatic neutral lipids. Hepatic relative mRNA expression of genes codifying for (L) *Lxrα* and its target genes involved in (M) cholesterol excretion and (N) de novo lipogenesis, oxysterols (O) synthesis and (P) catabolism, (Q) cholesterol synthesis and (R) uptake. Data are expressed as mean ± SEM. Comparison between two different groups was performed using the Mann-Whitney U Test (*p < 0.05; **p < 0.01; ***p < 0.001)

To explore the effects of WD feeding on the development of hepatocellular carcinoma, we injected wild-type mice with diethylnitrosamine (DEN) at 15 days of age. Then we fed them either a normal chow diet (CD) or a WD for eleven months. WD-fed mice presented an increased number of liver tumours per mouse, especially of small size (less than 0.5 cm of diameter), compared to mice fed CD (Fig. 1A-C). The tumours harvested from WD-fed mice showed an increased level of Ki67 and of Cyclin D1 mRNA (*Ccnd1*), two proliferation markers whose expression is usually enhanced in HCC tissues (Fig. 1D-F). The HE staining revealed marked steatosis in mice fed WD (Fig. 1G), which is accompanied by increased inflammation and fibrosis (Fig. 1H-J), two hallmarks of steatohepatitis, a predisposing condition for HCC development. Specifically, Tumour Necrosis Factor α (*Tnfα*), Interleukin 6 (Il6), and Transforming Growth Factor β (*Tgfβ*), whose expression is detected in MASH individuals and correlates with the disease progression [21–23], are induced in animals fed WD (Fig. 1H). Of note, TGFβ signalling is required for hepatic stellate cell activation and liver fibrogenesis, the latter being also induced in WD-fed animals, as indicated by Collagen 1a1 (*Col1a1*) expression and Sirius Red staining (Fig. 1I-J).

The analysis of neutral lipids revealed an extensive accumulation of cholesteryl esters (CE) and triglycerides in the tumour specimens of animals fed a WD (Fig. 1K). These results prompted us to investigate the expression profile of those genes involved in lipid metabolism. In the liver, Lxr can regulate cholesterol metabolism and the synthesis of new fatty acids. Intriguingly, in tumour tissue of WD-fed mice, while the expression of *Nr1h3* was significantly induced (Fig. 1L), the mRNA levels of its target genes involved in cholesterol excretion *Abcg5/8* and de novo lipogenesis (*Srebp1c, Fasn, Scd1)* were not changed between the two groups of mice (Fig. 1M-N). A possible reason for this net uncoupling between Lxr activity and high lipid content may be due to the reduced oxysterols availability. To this end, we evaluate the expression of genes involved in oxysterol synthesis (Cytochrome P450 27A1 and 46a1, *Cyp27a1* and *Cyp46a1*) and sulfation and excretion (Sulfotransferase 2B1, *Sult2b1*; ATP Binding Cassette, *Abcc1*) [24]. Interestingly, we found a significantly decreased oxysterol synthesis coupled with increased sulfation and secretion (Fig. 1O-P), which overall determine a reduction of oxysterol availability and consequent inactivation of Lxr in cancer specimens, despite its mRNA induction. Finally, we observed a net reduction in the expression of all those genes involved in cholesterol synthesis (Fig. 1Q), probably suggesting that an increased uptake of cholesterol and fatty acids, rather than an increased synthesis, is behind the high lipid levels observed. Of note, *Ldlr* expression did not change (Fig. 1R).

*Western diet feeding increases intestinal Lxr transcriptome.*

**Fig. 2:**
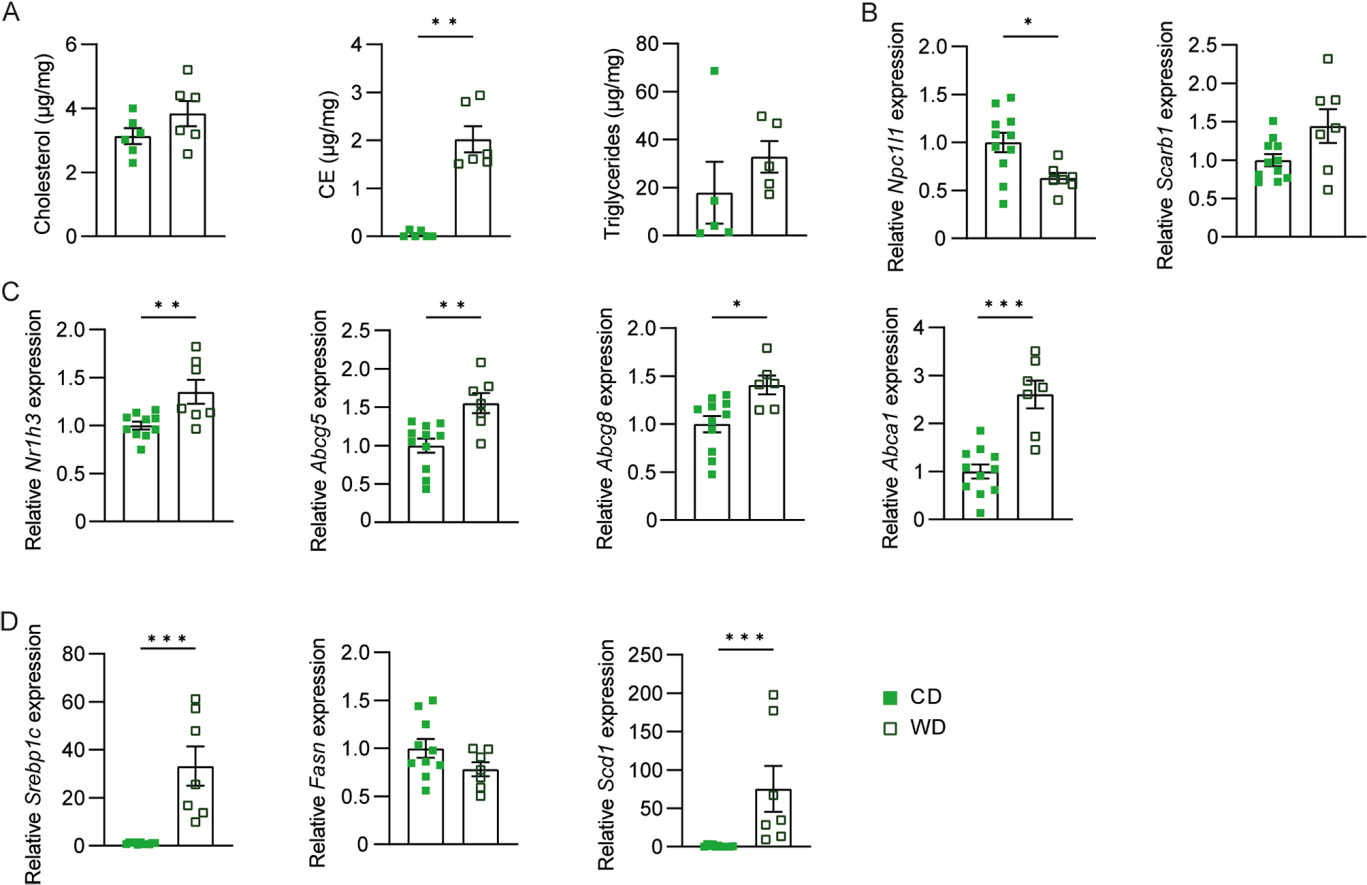
**Western diet feeding increases intestinal Lxr transcriptome.** Fifteen-day-old Wild Type C57BL6/J mice were injected with diethyl-nitrosamine (DEN) and then fed either a chow diet (CD) or a Western diet (WD) starting from one month of age. Mice were sacrificed after 12 months (*n* = 7–12 animals/group). (A) Intestinal cholesterol, cholesteryl esters, and triglycerides level. (B) Intestinal relative mRNA expression of cholesterol importer. (C) Intestinal relative mRNA expression of *Lxrα* and its target genes involved in cholesterol excretion. (D) Intestinal relative mRNA expression of Lxrα target genes involved in de novo lipogenesis. Data are expressed as mean ± SEM. Comparison between two different groups was performed using the Mann-Whitney U Test (*p < 0.05; **p < 0.01)

Several pieces of evidence suggested that changes in the intestinal epithelium may influence the dietary lipid uptake and disposal, therefore influencing the hepatic lipid accumulation [25, 26]. Hence, we explored whether the increased cholesteryl esters and triglyceride levels observed in WD-fed animals were dependent on enterocyte modifications. We observed a trend of increase in cholesterol and triglyceride accretion and a significant rise in the level of CE in the intestine (Fig. 2A). We next evaluate the expression level of the two major lipid importers, *Npc1l1* and Scavenger Receptor B1 (*Scarb1*) in the enterocytes. While the first was significantly downregulated upon WD feeding, the latter was only mildly affected by diet (Fig. 2B). Since the *in vivo* activation of LXR may decrease the NPC1L1 mRNA levels in the enterocytes [27], we then assessed the mRNA level of Lxr and its target genes involved in cholesterol excretion and de novo lipogenesis, finding an overall increase in mice fed WD (Fig. 2C-D). This indicates a major involvement in fatty acid synthesis and disposal in WD-fed animals:* Srebp1*c and *Scd1* are greatly induced, and, despite no changes in the mRNA level of *Fasn* being detected, we cannot exclude an increase in its enzymatic activity. Overall, these data suggest that the intestinal activation of Lxr correlates with an increased number of hepatic tumours.

*The intestinal activation of LXRα does not influence liver tumours under chow diet feeding.*

**Fig. 3:**
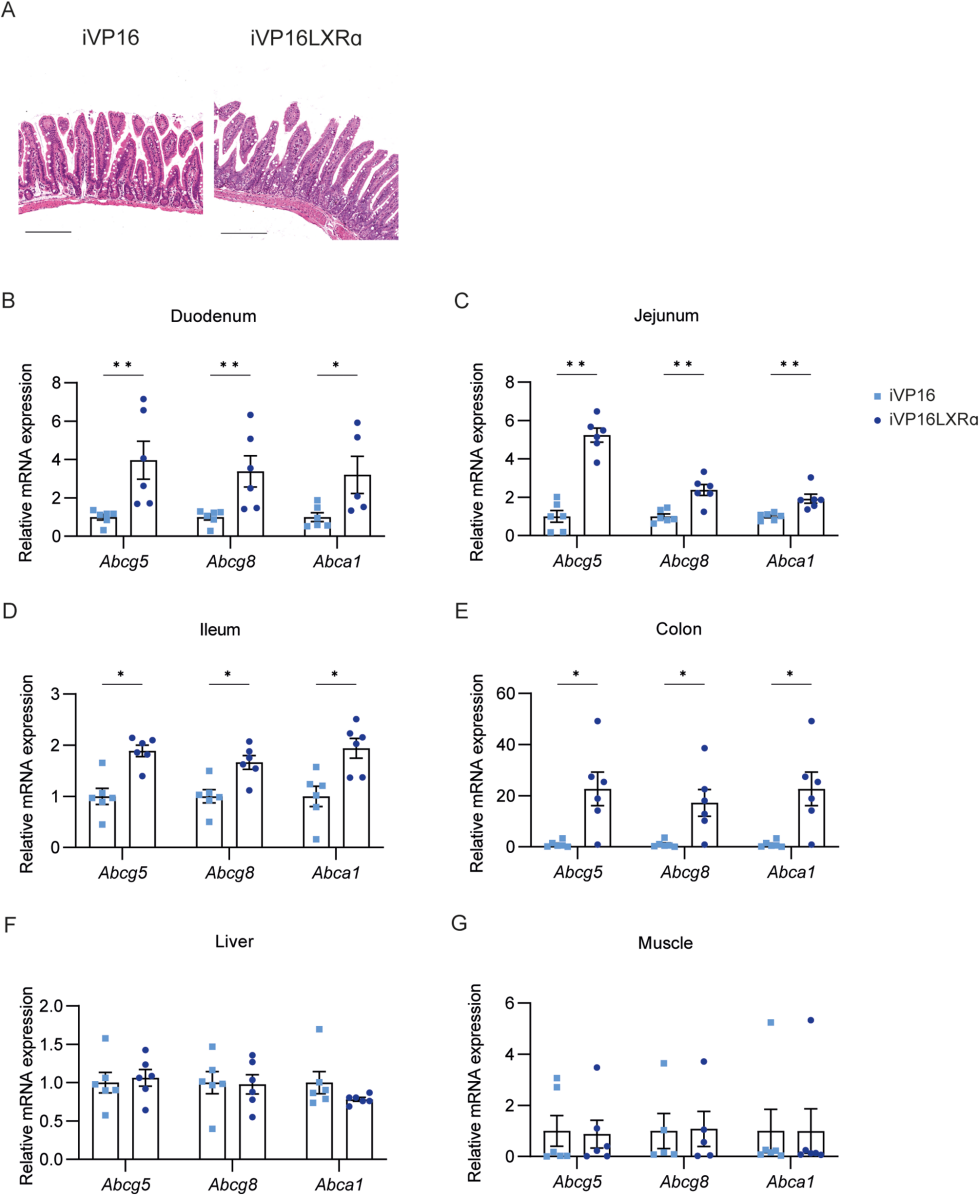
**iVP16LXRα mice display a specific intestinal induction of LXRα and its transcriptome.** iVP16LXRα and iVP16 control mice were fed a chow diet for 10 months (*n* = 6 mice/group). (A) Haematoxylin-Eosin (HE) of intestinal section (scale bar 200 μm). Relative Lxrα target genes expression involved in cholesterol excretion in (B) duodenum, (C) jejunum, (D) ileum, (E) colon, (F) liver, and (G) muscle. Data are expressed as mean ± SEM. Comparison between two different groups was performed using the Mann-Whitney U Test (*p < 0.05; **p < 0.01)

To understand if the intestinal chronic activation of Lxr was able to promote hepatic cancer, we took advantage of iVP16LXRα mice, in which the constitutively activated form of human LXRα (VP16LXRα) was under the control of the enterocyte-specific *Villin* promoter [19]. iVP16 mice were used as controls. No major modifications were detected in the intestinal architecture of the two genotypes at the time of sacrifice (Fig. 3A). The constitutive activation of LXRα, as indicated by the expression of its target genes, was highly induced in all the intestinal tracts of iVP16LXRα mice. Still, no changes were found in the liver and the muscle, two major metabolic organs (Fig. 3B-G). Altogether, these data indicate a specific intestinal activation of LXRα in iVP16LXRα mice compared to controls.

**Fig. 4:**
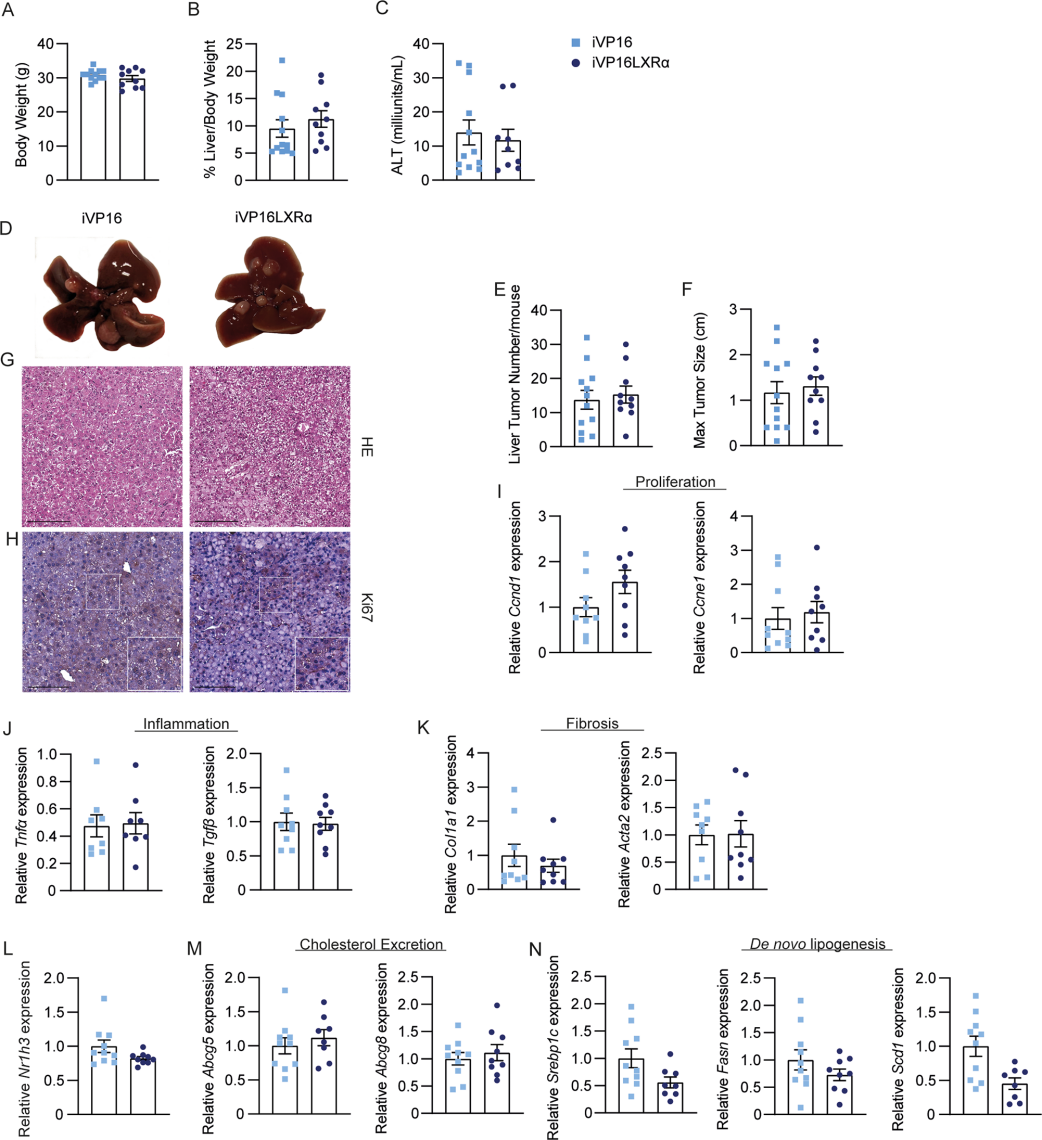
**The intestinal activation of LXRα does not influence liver tumours under chow diet feeding.** Fifteen-day-old iVP16LXRα and iVP16 control mice were injected with diethyl-nitrosamine (DEN) and fed a chow diet (CD) starting from one month of age. Mice were sacrificed after 12 months (*n* = 10–12 animals/group). (A) Body weight. (B) Liver to body weight ratio. (C) Alanine Aminotransferase level (ALT). (D) Gross morphology of the liver at the moment of sacrifice. (E) The average number of tumours per mouse. (F) Maximal tumour diameter. Liver tumour sections stained with (G) Haematoxylin- Eosin (HE) or (H) proliferation marker Ki-67 (scale bar 200 μm). Relative mRNA expression of genes involved in (I) proliferation, (J) inflammation, and (K) fibrosis in liver tumour specimens. Relative mRNA expression of (L) Lxrα and its target genes involved in (M) cholesterol excretion and (N) de novo lipogenesis in liver tumour specimens. Data are expressed as mean ± SEM. Comparison between two different groups was performed using the Mann-Whitney U Test

To explore if intestinal chronic Lxrα activation plays a pivotal role in HCC development, we compared DEN-induced hepatocarcinogenesis in iVP16LXRα mice and iVP16 controls fed a normal chow diet for eleven months. No changes in body weight gain, liver-to-body weight ratio and alanine aminotransferase (ALT) levels were detected between the two genotypes (Fig. 4A-C). The macroscopic analysis of the liver revealed no difference in the tumour number and dimension (Fig. 4D-F). Accordingly, no differences were detected in the hepatic parenchyma and the level of proliferation markers (Fig. 4G-I), nor in markers of inflammation and fibrosis, as indicated by *Tnfα* and *Tgfβ* as well as *Col1a1* and *Acta2* expression, respectively (Fig. 4J-K). Intriguingly, the expression of genes involved in lipid metabolism, both cholesterol and fatty acids, was unchanged between the two genotypes (Fig. 4L-N) and no differences were detected in the fatty acids composition in plasma (Table 3). Despite no changes in the neutral lipids were observed in the intestine (Supplementary Fig. 1A), in the ileum of iVP16LXRα mice we detected an increase in monounsaturated fatty acids (MUFA), especially palmitoleic acid (C16:1 n-9) and oleic acid (C18:1 n-9), while the palmitic acid (C16:0) was reduced (Table 4); these data are probably correlated with the induction of intestinal Scd1, a target of Lxrα. Also, the hepatic level of neutral lipid does not change between the two genotypes (Supplementary Fig. 1B), while saturated fatty acid (SFA) specimens are lower in iVP16LXRα mice than in iVP16 ones (Table 5). All these data suggest that during chow diet feeding the overexpression of intestinal Lxrα only partially influences the hepatic lipid metabolism, but this is not sufficient to drive HCC onset.

**Table 3:** **Plasmatic fatty acids composition.** Data are expressed as mean ± SEM of the mass percentage measured in the plasma of iVP16 and iVP16LXRα mice treated with DEN and fed with either chow or western diet (*n* = 6 per group). Comparison between genotypes fed with the same diet was performed using the Mann-Whitney U Test (*p<0.05; **p < 0.01)

	Chow Diet		Western Diet	
Fatty Acids	iVP16	iVP16LXRα	iVP16	iVP16LXRα
C14:0	0.898 ± 0.152	0.962 ± 0.155	1.61 ± 0.151	0.972 ± 0.094**
C16:0	27.114 ± 1.243	26.273 ± 0.923	28.7 ± 1.326	25.672 ± 0.179
C18:0	15.854 ± 1.348	16.356 ± 1.535	17.947 ± 2.053	13.619 ± 0.887
C24:0	1.891 ± 0.328	1.921 ± 0.167	1.12 ± 0.159	1.934 ± 0.095**
C14:1 n-7	0.261 ± 0.066	0.32 ± 0.047	0.453 ± 0.069	0.262 ± 0.031*
C16:1 n-9	0.167 ± 0.014	0.191 ± 0.015	0.13 ± 0.011	0.177 ± 0.011*
C16:1 n-7	1.864 ± 0.173	1.498 ± 0.096	3.56 ± 0.35	3.17 ± 0.158
C18:1 n-9c	16.859 ± 1.517	16.23 ± 0.596	24.217 ± 0.394	22.246 ± 0.47*
C18:1 n-7c	2.433 ± 0.245	2.146 ± 0.164	2.547 ± 0.135	3.016 ± 0.187
C20:1 n-9	0.193 ± 0.017	0.198 ± 0.009	0.22 ± 0.008	0.194 ± 0.013
C16:2 n-6	0.125 ± 0.031	0.158 ± 0.015	0.267 ± 0.008	0.227 ± 0.007**
C18:2 n-6c	20.837 ± 2.255	21.75 ± 1.711	12.103 ± 2.164	13.708 ± 0.368
C18:3 n-6	0.714 ± 0.132	0.458 ± 0.059	0.453 ± 0.046	0.308 ± 0.026*
C20:3 n-6	0.678 ± 0.107	0.792 ± 0.094	0.787 ± 0.151	1.329 ± 0.061**
C20:4 n-6	10.106 ± 1.738	10.739 ± 0.939	5.878 ± 0.91	13.159 ± 0.805**
%SFA	45.759 ± 2.394	45.514 ± 2.212	49.378 ± 3.342	42.199 ± 1.057
%MUFA	21.779 ± 1.725	20.586 ± 0.65	31.13 ± 0.54	29.067 ± 0.749*
%PUFA	32.461 ± 3.999	33.9 ± 2.573	19.49 ± 3.122	28.733 ± 0.943**

**Table 4:** **Ileal fatty acids composition.** Data are expressed as mean ± SEM of the mass percentage measured in the ileum of iVP16 and iVP16LXRα mice treated with DEN and fed with either chow or western diet (*n* = 6 per group). Comparison between genotypes fed with the same diet was performed using the Mann-Whitney U Test (*p<0.05; **p<0.01)

		Chow Diet		Western Diet		
Fatty Acids		iVP16	iVP16LXRα	iVP16	iVP16LXRα	
C14:0	1.077 ± 0.206	0.805 ± 0.246	2.136 ± 0.321	2.178 ± 0.223		
C16:0	26.406 ± 1.598	22.347 ± 1.743*	22.409 ± 4.639	18.875 ± 3.889		
C18:0	18.81 ± 2.283	10.887 ± 3.065	13.211 ± 3.891	7.281 ± 1.272		
C20:0	0.43 ± 0.056	0.312 ± 0.073	0.327 ± 0.066	0.216 ± 0.036		
C24:0	0.972 ± 0.181	0.593 ± 0.177	0.383 ± 0.169	0.248 ± 0.038		
C14:1 n-7	0.276 ± 0.035	0.147 ± 0.053	0.333 ± 0.035	0.354 ± 0.027		
C16:1 n-9	0.148 ± 0.034	0.372 ± 0.051**	5.421 ± 5.083	5.22 ± 4.546		
C16:1 n-7	2.76 ± 0.557	2.966 ± 0.522	7.685 ± 1.108	8.475 ± 0.947		
C18:1 n-9c	22.54 ± 1.344	28.907 ± 1.541*	36.64 ± 3.625	46.949 ± 1.822*		
C18:1 n-7c	2.58 ± 0.228	2.408 ± 0.116	2.765 ± 0.343	3.074 ± 0.178		
C20:1 n-9	0.334 ± 0.027	0.528 ± 0.051*	0.347 ± 0.019	0.32 ± 0.024		
C16:2 n-6	0.161 ± 0.018	0.176 ± 0.013	0.245 ± 0.012	0.214 ± 0.007*		
C18:2 n-6c	16.61 ± 2.169	25.232 ± 2.823	4.921 ± 0.723	4.497 ± 0.129		
C18:3 n-6	0.371 ± 0.063	0.338 ± 0.103	0.177 ± 0.044	0.095 ± 0.02		
C20:2 n-6	0.39 ± 0.049	0.293 ± 0.041	0.248 ± 0.075	0.16 ± 0.016		
C20:3 n-6	0.88 ± 0.167	0.592 ± 0.142	0.405 ± 0.179	0.242 ± 0.036		
C20:4 n-6	4.631 ± 0.908	2.702 ± 0.797	2.083 ± 0.931	1.453 ± 0.229		
C22:4 n-6	0.498 ± 0.089	0.297 ± 0.078	0.184 ± 0.087	0.114 ± 0.017		
C22:6 n-3	0.118 ± 0.026	0.092 ± 0.019	0.072 ± 0.031	0.027 ± 0.005		
%SFA	47.697 ± 3.828	34.946 ± 4.712	38.468 ± 3.883	28.799 ± 4.203		
%MUFA	28.64 ± 1.864	35.329 ± 2.086*	53.193 ± 4.36	64.395 ± 4.482		
%PUFA	23.661 ± 3.126	29.724 ± 3.178	8.338 ± 1.991	6.805 ± 0.432		

**Table 5:** **Hepatic fatty acids composition.** Data are expressed as mean ± SEM of the mass percentage measured in the liver of iVP16 and iVP16LXRα mice treated with DEN and fed with either chow or western diet (*n* = 6 per group). Comparison between genotypes fed with the same diet was performed using the Mann-Whitney U Test (*p<0.05; **p<0.01)

	Chow Diet		Western Diet	
Fatty Acids	iVP16	iVP16LXRα	iVP16	iVP16LXRα
C14:0	0.903 ± 0.093	0.462 ± 0.055**	1.028 ± 0.031	0.718 ± 0.07**
C16:0	26.43 ± 0.916	23.435 ± 1.286	24.057 ± 0.98	25.084 ± 0.998
C18:0	16.3 ± 1.698	10.973 ± 1.185*	7.615 ± 1.811	8.661 ± 1.52
C24:0	1.793 ± 0.147	2.104 ± 0.225	1.186 ± 0.198	0.694 ± 0.176
C14:1 n-7	0.249 ± 0.036	0.128 ± 0.021	0.222 ± 0.009	0.134 ± 0.014**
C16:1 n-9	0.443 ± 0.103	0.505 ± 0.069	0.636 ± 0.165	0.914 ± 0.24
C16:1 n-7	2.56 ± 0.695	1.682 ± 0.164	5.133 ± 0.909	3.363 ± 0.35
C18:1 n-9c	25.595 ± 2.59	29.165 ± 1.246	42.985 ± 1.81	46.96 ± 1.627
C18:1 n-7c	3.542 ± 0.398	4.342 ± 0.789	5.634 ± 0.438	5.864 ± 0.278
C20:1 n-9	0.359 ± 0.038	0.933 ± 0.336**	0.816 ± 0.12	1.015 ± 0.084
C16:2 n-6	0.167 ± 0.016	0.097 ± 0.014*	0.177 ± 0.017	0.107 ± 0.014*
C18:2 n-6c	13.922 ± 1.619	17.697 ± 0.903	5.726 ± 0.305	3.451 ± 0.457**
C18:3 n-6	0.49 ± 0.074	0.386 ± 0.092	0.282 ± 0.053	0.187 ± 0.036
C18:3 n-3	0.167 ± 0.053	0.201 ± 0.057	0.033 ± 0.011	0.08 ± 0.009*
C20:2 n-6	0.558 ± 0.123	0.749 ± 0.111	0.682 ± 0.084	0.528 ± 0.088
C20:3 n-6	0.753 ± 0.052	1.157 ± 0.084**	0.523 ± 0.034	0.28 ± 0.078*
C20:4 n-6	5.113 ± 0.636	5.28 ± 0.738	2.956 ± 0.308	1.821 ± 0.476
C22:4 n-6	0.477 ± 0.149	0.502 ± 0.101	0.211 ± 0.048	0.103 ± 0.036
C22:6 n-3	0.172 ± 0.027	0.193 ± 0.03	0.09 ± 0.012	0.028 ± 0.01**
%SFA	45.426 ± 2.506	36.974 ± 2.383*	33.888 ± 2.77	35.158 ± 2.357
%MUFA	32.75 ± 3.17	36.757 ± 2.243	55.428 ± 3.062	58.253 ± 1.7
%PUFA	21.822 ± 2.24	26.267 ± 0.728	10.683 ± 0.383	6.589 ± 1.138*

*The intestinal activation of LXRα promotes liver tumours under Western diet feeding.*

**Fig. 5:**
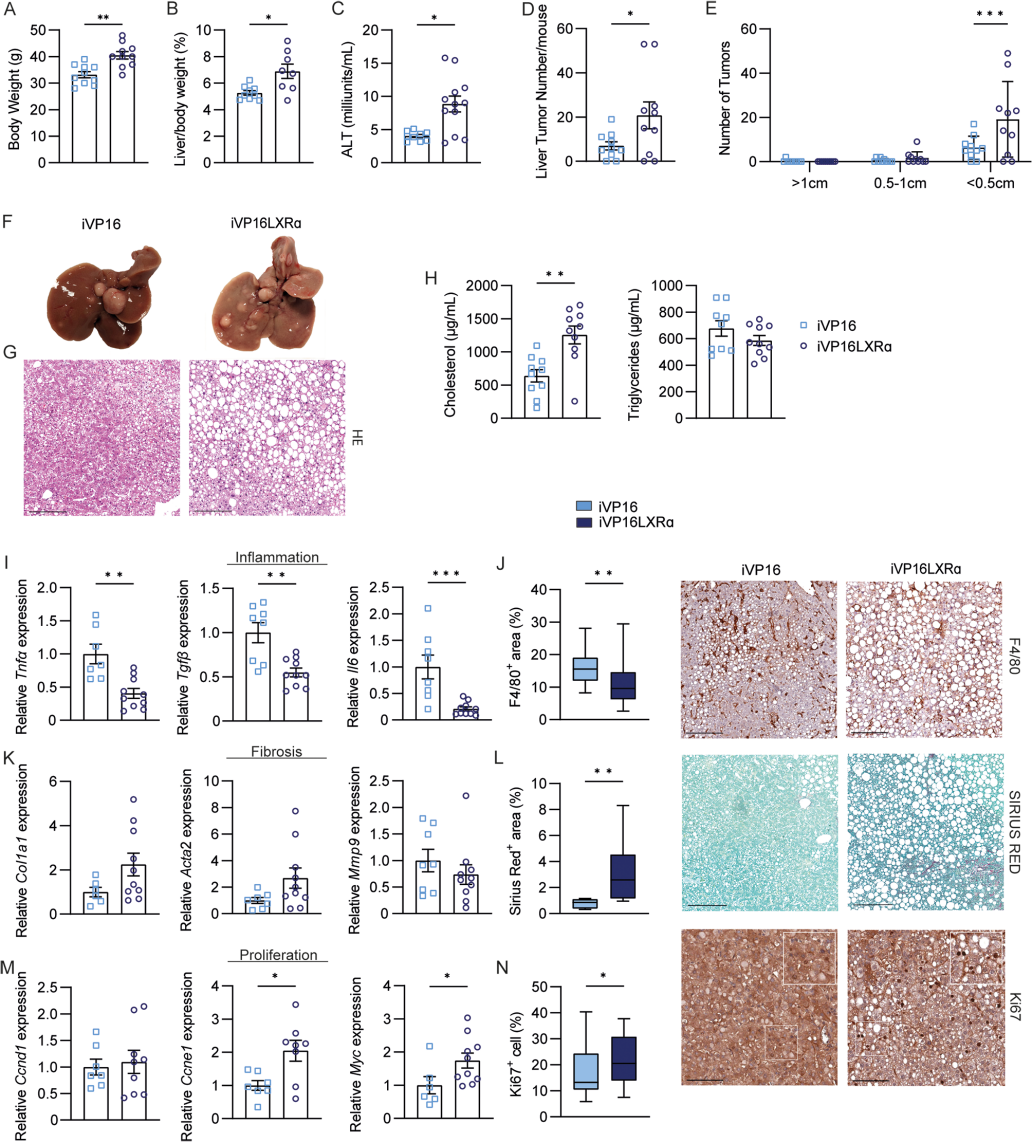
**The intestinal activation of LXRα promotes liver tumours under Western diet feeding**. Fifteen-day-old iVP16LXRα and iVP16 control mice were injected with diethyl-nitrosamine (DEN) and fed a western diet starting from one month of age. Mice were sacrificed after 8 months (*n* = 10 animals/group). (A) Body weight. (B) Liver to body weight ratio. (C) Alanine Aminotransferase level (ALT). (D) The average number of tumours per mouse. (E) The average number of tumours per mouse based on tumour diameter cut-off. (F) Gross morphology of the liver at the moment of sacrifice. (G) Haematoxylin-Eosin (HE) staining of liver tumour sections (scale bar 200 μm). (H) Circulating neutral lipids. (I) Hepatic relative mRNA expression of inflammatory genes. (J) F4/80 staining (scale bar 200 μm) and quantification of liver tumour sections. (K) Hepatic relative mRNA expression of fibrotic genes. (L) Sirius Red staining (scale bar 200 μm) and quantification of liver tumour sections. (M) Hepatic relative mRNA expression of proliferation markers. (N) Ki67 staining (scale bar 100 μm) and quantification of liver tumour sections. Data are expressed as mean ± SEM. Comparison between two different groups was performed using the Mann-Whitney U Test (*p < 0.05; **p < 0.01; ***p < 0.001)

To test whether the intestinal chronic activation of Lxrα can induce liver tumours in the presence of an increased lipids consumption, such as one typical of WD, iVP16LXRα mice and iVP16 controls were injected with DEN and then fed WD for seven months. Intriguingly, prolonged exposure to WD, similar to those used in previous experiments (11 months), resulted in cachexia and early death in iVP16LXRα mice (5/10 mice), but not in controls. After 7 months of diet, iVP16LXRα mice gained more weight than controls (Fig. 5A). Likewise, iVP16LXRα mice displayed an increase in the liver-to-body weight ratio and the ALT level (Fig. 5B-C). HCC incidence was numerically higher in iVP16LXRα mice, with an increase in small-size tumours (less than 0.5 cm in diameter) (Fig. 5D-F). Of note, the livers harvested from iVP16LXRα mice were more pale compared to iVP16 controls, indicative of an increase in lipid accretion, confirmed also by the accumulation of lipid droplets in the hepatic parenchyma, clear in the HE staining (Fig. 5F-G). Plasma analysis indicated a doubled circulating cholesterol content in iVP16LXRα mice compared to controls. In contrast, no changes were detected in the triglycerides level (Fig. 5H). Interestingly, iVP16LXRα mice displayed a different circulating fatty acids composition compared to iVP16 mice (Table 3), especially of unsaturated species. However, no differences were detected in the intestinal neutral lipid amount (Supplementary Fig. 2) and only small changes were detected in the ileum of these mice, with a consistent increase of oleic acid (C18:1n9) (Table 4). Despite the marked steatosis, iVP16LXRα mice did not present an increased hepatic inflammatory signature, as indicated by the halved expression of Tumour Necrosis Factor α (*Tnfα*), Tumour Growth Factor β (*Tgfβ*) and Interleukin 6 (*Il6*) as well as the decreased level of the macrophage marker F4/80 (Fig. 5I-J). Moreover, although the markers of fibrosis were only slightly increased in iVP16LXRα mice (Fig. 5K), they displayed an increased collagen deposition as indicated by the Sirius Red staining (Fig. 5L). Finally, the proliferative markers Cyclin E1 (*CcnE1*) and *Myc* were induced in hepatic tumours harvested from iVP16LXRα mice, while no differences were detected in *Ccnd1* expression levels (Fig. 5M). The Ki67 staining showed a trend to increase in iVP16LXRα mice compared to controls (Fig. 5N). Therefore, the chronic activation of intestinal Lxrα exacerbates hepatocarcinogenesis only in the presence of a high amount of dietary lipids.

*The cholesterol accumulation is uncoupled from the Lxr transcriptome in the liver tumours of iVP16LXRα mice.*

**Fig. 6:**
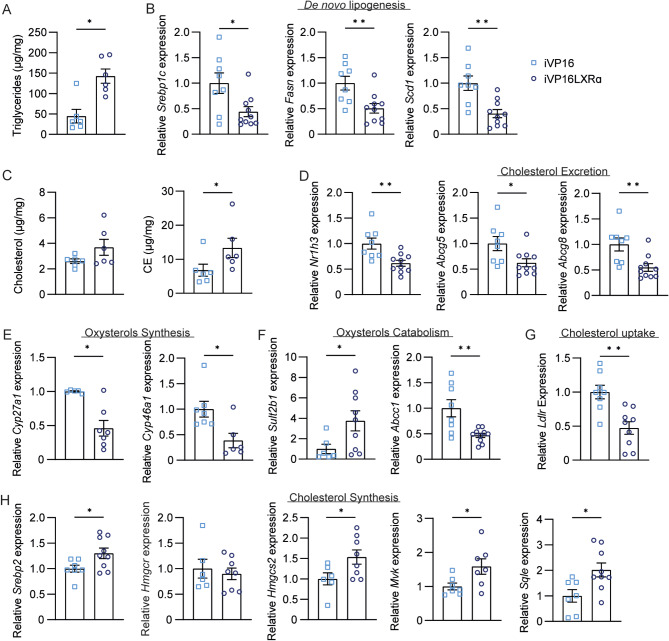
**The cholesterol accumulation is uncoupled from the Lxr transcriptome in the liver tumour of iVP16LXRα mice.** Fifteen-day-old iVP16LXRα and iVP16 control mice were injected with diethyl-nitrosamine (DEN) and fed a western diet starting from one month of age. Mice were sacrificed after 8 months (*n* = 10 animals/group). (A) Hepatic triglycerides level. (B) Relative mRNA expression of de novo lipogenesis in liver tumour specimens. (C) Hepatic cholesterol and cholesterol-esters (CE) level. (D) Relative mRNA expression of *Lxrα* and its target genes involved in cholesterol excretion in liver tumour specimens. Relative mRNA expression of genes involved in oxysterol (E) synthesis and (F) catabolism as well as in cholesterol (G) uptake and (H) synthesis in liver tumour specimens. Data are expressed as mean ± SEM. Comparison between two different groups was performed using the Mann-Whitney U Test (*p < 0.05; **p < 0.01)

To fully examine the correlation between the WD feeding and intestinal Lxrα underlining an increase in liver tumour vulnerability, we analysed the hepatic lipid metabolism. In line with the results obtained in WT mice, we found that the intestinal activation of Lxrα correlates with an increased accumulation of hepatic triglycerides (Fig. 6A), and a reduction of the relative abundance of polyunsaturated fatty acids specimens (Table 5), thus correlating with the more pronounced liver steatosis observed in iVP16LXRα. However, the expression level of genes involved in de novo lipogenesis is halved in iVP16LXRα mice compared to iVP16 ones (Fig. 6B). Also, the amount of hepatic cholesterol and CE are increased in iVP16LXRα (Fig. 6C), but they do not correlate with the mRNA level of Lxrα and its target genes, which is less expressed in iVP16LXRα mice than in controls (Fig. 6D). Intriguingly, the genes involved in oxysterol synthesis are significantly lower expressed in the iVP16LXRα mice than in iVP16 ones (Fig. 6E), together with a boost of *Sultb1* expression and a downregulation of *Abcc1* in iVP16LXRα animals (Fig. 6F). While *Sultb1* is involved in oxysterols catabolism, *Abcc1* regulates their excretion. These data suggest a net decrease in Lxrs ligands disposal in the liver of iVP16LXRα mice, possibly correlated with the significative reduction of *Ldlr* expression (Fig. 6G). Despite this net uncoupling between cholesterol accumulation and Lxr transcriptome in the liver tumours of iVP16LXRα mice, these animals showed an induction of the genes involved in the cholesterogenesis pathway, especially of Squalene Epoxidase (*Sqle*), whose overexpression in MASH-HCC tumours is associated with poor patient outcomes [28, 29], whereas no changes were detected in the mRNA level of *Hmgcr* (Fig. 6H).

*LXR transcriptome is downregulated in human HCC and correlates with low survival.*

**Fig. 7:**
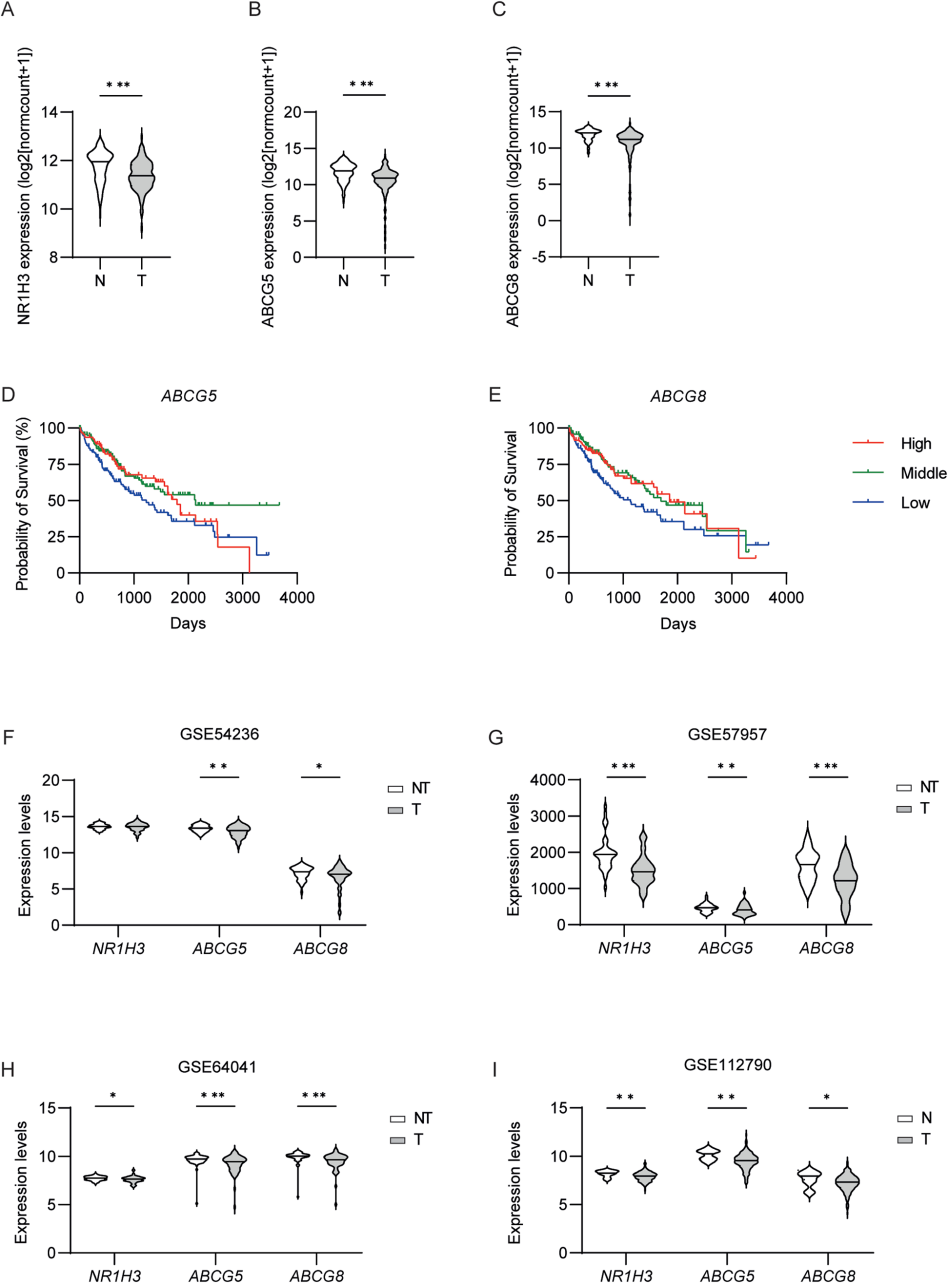
**LXR transcriptome is downregulated in human HCC and correlates with low survival.** (A-C) Bioinformatics analysis of the expression of LXR and its target genes in human healthy liver (N, *n* = 109) and tumour tissue (T, *n* = 369) using TCGA TARGET GTEx and UCSC Xena browser. (D-E) Kaplan-Meier curve of survival of patients with HCC and high-, middle- or low-expression levels of ABCG5 and ABCG8 as determined by TCGA data sets. (F-I) Expression of LXR and its target genes in GEO dataset of human liver cancer. N: Normal liver, NT: Non Tumoral Liver, T: Liver Tumor. Comparison between two different groups was performed using the Mann-Whitney U Test (*p<0.05; **p<0.01; ***p < 0.001)

Our data collected in mice showed a net uncoupling between cholesterol accumulation in the liver and the expression of the Lxr transcriptome. Since cholesterol accumulation is a major characteristic of human HCC [30], we wanted to determine the clinical significance and prognostic value of LXRα transcriptome in human HCC. It is important to note that, despite its aetiology, HCC is considered a metabolic cancer: the consumption of an unhealthy diet together with a sedentary lifestyle can lead to an increased Body Mass Index (BMI) that positively correlates with liver cancer development and mortality, even in subjects with hepatitis B or C [31, 32]. We analysed the mRNA expression of LXRα and its target genes using TCGA (The Cancer Genome Atlas) datasets. Our results show that the expression of *NR1H3* was significantly downregulated in HCC tissues compared to healthy ones (Fig. 7A). Correspondingly, we observed also a reduction in the expression of LXR target genes related to cholesterol metabolism, namely *ABCG5* and *ABCG8* (Fig. 7B-C). The Kaplan-Meier survival analysis revealed that the low expression level of *ABCG5* and *ABCG8* -possible due to a decreased activation of LXR- correlates with a decreased survival probability in humans with HCC (*p* = 0.0253 and *p* = 0.0325, respectively; Fig. 7D-E). To corroborate the data, we also evaluate the expression of *NR1H3, ABCG5* and *ABCG8* in four different GEO datasets of human liver cancer, observing a significant reduction of their level in tumour tissue compared to non-tumoral one (Fig. 7F-I). Altogether, these data suggested that in human HCC patients, LXR activation is uncoupled from the cholesterol level, and may predict a worse prognosis.

## Discussion

Dietary intake is well recognized as a crucial factor in preserving metabolic health [33]. Indeed, an increase in energy introit has been correlated with obesity and metabolic diseases, including metabolic dysfunction-associated fatty liver disease and type 2 diabetes mellitus, which represent the primary aetiology of cancer [34]. Importantly, not only the caloric intake per se but also the quality of the fats present in the diet plays a role in the development of MASH and HCC. Using mouse models, it has been demonstrated that a diet enriched in fatty acids is not able to drive the progression from metabolic diseases to HCC, but that cholesterol has to be present to generate a lipotoxic and pro-inflammatory environment that leads to aberrant cell proliferation [20]. In response to overnutrition, hepatocytes accumulate triglycerides, cholesterol and CE, affecting and altering the functions of subcellular organelles and membranes that collectively impair cellular metabolism and behaviour [35, 36]. A large flux of dietary cholesterol occurs daily between the gut and the liver, and dietary cholesterol-induced MASH-HCC formation was associated with intestinal modifications [25, 26, 37].

Here, we showed that a Western diet regimen, with increased content of cholesterol and sugars, induces the development of HCC despite the upregulation of Lxrα and its target genes in the intestine. To validate these results we took advantage of a mice model in which Lxrα is chronically activated in the intestine and we observed that while it appears dispensable during a normal chow diet, it is crucial to drive HCC in the presence of metabolic conditions as the ones induced by WD feeding. Specifically, intestinal Lxrα activation promotes lipid accumulation in the liver, increasing the cellular energy disposal to sustain the rapid cell proliferation peculiar to cancer. Whether the establishment of a MASH scenario before the induction of tumours via DEN injection would promote the same results was not assessed in this study, although it could have brought new information in the comprehension of the disease’s progression.

Whole body LXRs knockout mice fed a chow diet showed a decreased fat storage in the liver and the WAT with an increase in futile energy dissipation in major metabolic organs; by contrast, the presence of Lxrs resulted in reduced hepatic cholesterol but increased fat storage in the liver and the WAT [38]. Our results supported the hypothesis that Lxrα in the gut is sufficient to sustain the major metabolic modifications described, while the hepatic Lxrs activity appears less important. Indeed, in mice in which the intestinal Lxrα is active, there is a net increase of triglycerides and cholesteryl esters in the liver, while the cholesterol level does not change significantly.

The activation of Lxrs in the gut, either induced by the diet or the genetic one, drives a similar tumoural phenotype. However, differently from WT mice fed WD, iVP16LXRα mice display a consistent reduction of inflammation. We cannot exclude that, like other nuclear receptors [39], Lxr might drive a gut-liver novel metabolic pathway where other signals coming from the intestine, either enterokines produced by the gut itself or byproducts of the microbiota, contribute to the phenotype we observed.

Our previous results showed that the intestinal chronic activation of Lxrα was sufficient to increase cholesterol excretion and protect the liver from lipid accumulation under a diet enriched in cholesterol [19]. These data, obtained in a non-tumoral scenario, do not correlate the activity of intestinal Lxrα with that of the hepatic ones. By contrast, here we illustrate that in a carcinogenic background, there is a net uncoupling between the intestinal Lxrα activation and the concomitant downregulation of hepatic Lxrα which promotes metabolic HCC. We excluded that the different diets used may influence the outcomes obtained since both WD and a high cholesterol diet with DEN resulted in a similar steatosis score with a comparable accumulation of hepatic cholesterol and triglycerides [40].

In a physiological context, the level of cholesterol is tightly regulated inside the cell. Indeed, while a low cholesterol concentration induces an increased hepatic cholesterol uptake and synthesis via SREBP2, a high cholesterol level promotes the activation of LXRs and the upregulation of all those genes involved in cholesterol excretion finally reducing the cellular cholesterol amount. During cancer, this equilibrium is disrupted: there is a net uncoupling between the levels of cholesterol and the LXRs activation. The neoplastic cell takes advantage of cholesterol disposal to sustain increased proliferation to support tumour growth [41]. To this end, the cancerous cell upregulates the acquirement of new cholesterol even if the cholesterol level is high, and concomitantly downregulates the LXRs pathway via a substantial reduction of oxysterols content [14]. Noteworthy, in the liver of our mice with increased intestinal Lxrα activity the oxysterols synthesis is halved with a concomitant increase in their excretion, thus corroborating the hypothesis that a high hepatic lipids disposal paradoxically correlates with a drastic decrease of oxysterols that finally results in lower activation of hepatic Lxrs pathway [24, 42, 43]. Indeed, we observed a decreased expression of genes involved in lipogenesis and cholesterol excretion regulated by Lxrα (Fig. 6). We also confirmed these data in humans, where HCC is usually characterized by an increased accumulation of cholesterol [30]. The LXR transcriptome is downregulated in HCC patients, and low levels of LXR target genes are associated with a decreased overall survival rate (Fig. 6). Unfortunately, due to ethical limitations, we do have not the possibility to explore whether this reduction is associated with an increased intestinal LXR activation.

In the scenario described here, the liver senses the reduction of intestinal lipids due to the constitutive activation of Lxrα and counteracts by maintaining a high level of intracellular lipids to support the HCC growth. Intriguingly, treatment with Sorafenib, an anticancer agent used to inhibit the neoplastic growth in HCC, binds and stabilizes LXRs, thus promoting its activation and partial tumour remission [16, 17]. However, the loss of LXRα strongly reduced the therapeutic efficacy of sorafenib, while LXRβ was irrelevant. Importantly, the ability to overcome therapy resistance in HCC was granted by the concomitant LXR activation and Raf-SCD1 inhibition by creating a lipotoxic environment [16]. Notable, the intestinal ablation of Scd1 increases steatohepatitis and HCC in a murine model, blunting the LXRs’ activity and decreasing the hepatic monounsaturated fatty acids level with an increase of cholesterol accumulation in the liver [25]. Our results showed an ileal-specific fatty acids profile with increased palmitoleic and oleic acid (Table 4) resembling the enterocyte activation of Scd1 under the constitutive induction of Lxrα [19, 44]; therefore, it would be interesting to evaluate if the inhibition of Scd1 concomitant with the increased activation of Lxrα in the gut may exert a protective effect towards the liver. This may eventually offer new therapeutic opportunities to limit MASH and metabolic-HCC, given that the enterocytes are more easily exposed to the drugs.

Our group has previously demonstrated that the activation of intestinal LXRα restrains colorectal cancer growth [11], thus leading to the question of whether the activation of intestinal LXRs could be beneficial or not for the whole organism. As described in our results, LXRs’ activation is dispensable in the presence of a regular standard diet. Still, it has to be carefully evaluated in those subjects in which the diet (especially a high-fat, high-cholesterol diet) plays a major role in the disease, such as obese and MASH patients.

Overall, our study demonstrated that, despite a similar genetic background, Western diet feeding boosts hepatic carcinogenesis, therefore drawing attention to the gut and its role in liver cancer metabolism. This establishes the relevance of the intestine in influencing the susceptibility to MASH-HCC and points to intestinal LXRα activation as a driver of the metabolic environment of liver cancer in the presence of high dietary cholesterol.

### Electronic supplementary material

Below is the link to the electronic supplementary material.

Supplementary Material 1

Supplementary Material 2

## Data Availability

Data and study material will be available to other researchers upon reasonable request to the corresponding author.

## Abbreviations

ALT: Alanine aminotransferase
ABCG5/8: ATP Binding Cassette 5 and 8
CE: Cholesteryl esters
CD: Chow diet
Ccnd1: Cyclin D1
CcnE1: Cyclin E1
DEN: Diethylnitrosamine
FASN: Fatty Acids Synthase
HCC: Hepatocellular carcinoma
Il6: Interleukin 6
LXRs: Liver X Receptors
LDLR: Low-Density Lipoprotein Receptor
MASH: Metabolic dysfunction-associated steatohepatitis
Mvk: Mevalonate Kinase
MUFA: Monounsaturated fatty acids
NPC1L1: Niemann-Pick C1-Like 1
MUFA: Polyunsaturated fatty acids
SFA: Saturated fatty acid
Scarb1: Scavenger Receptor B1
Sqle: Squalene Epoxidase
SCD1: Stearoyl-CoA desaturase-1
SREBP: Sterol regulatory element-binding protein
Tgfβ: Tumour Growth Factorβ
Tnfα: Tumour Necrosis Factorα
WD: Western diet
