# Supplementary material for: Pivotal role of intestinal cholesterol and nuclear receptor LXR in metabolic liver steatohepatitis and hepatocarcinoma
Source: Cell Biosci. 2024 Jun 1;14:69. doi: 10.1186/s13578-024-01248-y (PMC11144344; doi:10.1186/s13578-024-01248-y)
Supplement: Supplementary file 1 — Supplementary Material 1 [file 13578_2024_1248_MOESM1_ESM.pdf]

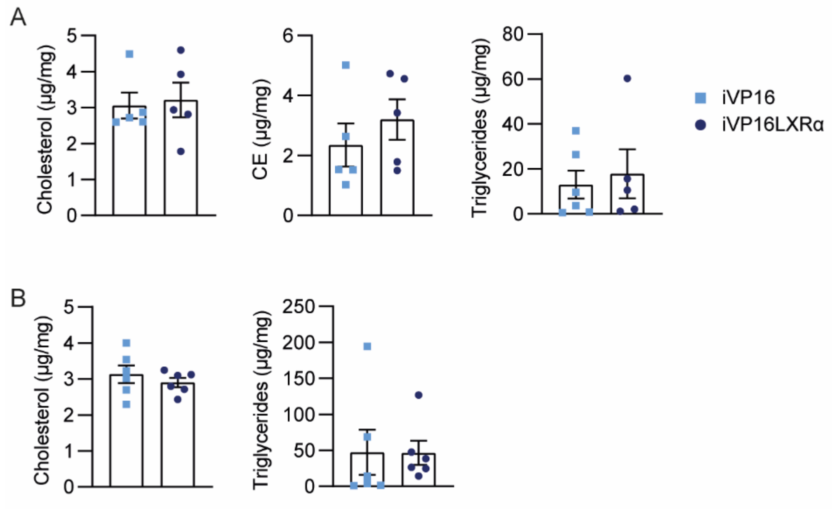

Supplementary Fig. 1 Neutral lipids in tumours of mice fed chow diet. Fifteen-day-old iVP16LXRα and iVP16 control mice were injected with diethyl-nitrosamine (DEN) and fed a chow diet (CD) starting from one month of age. Mice were sacrificed after 12 months (n=10-12 animals/group). (A) Hepatic cholesterol, cholesterol-esters (CE) and triglyceride level. (B) Intestinal cholesterol and triglyceride level. Data are expressed as mean±SEM. Comparison between two different groups was performed using the Mann-Whitney U Test.
